# Prediction of shock heating during ultrasound-induced bubble collapse using real-fluid equations of state

**DOI:** 10.1016/j.ultsonch.2023.106663

**Published:** 2023-11-02

**Authors:** Saeed Bidi, Armand Shams, Phoevos Koukouvinis, Manolis Gavaises

**Affiliations:** a School of Mathematics, Computer Sciences & Engineering, City, University of London, UK; b Institut Jean Le Rond D’Alembert, Sorbonne Université and CNRS UMR 7190, F-75005 Paris, France

**Keywords:** Bubble collapse dynamics, Real-liquid EoS, IAPWS data, MNASG, Tait EoS, Shock wave lithotripsy (SWL)

## Abstract

Numerical simulations of collapsing air bubbles considering complex and more accurate equations of state (EoS) for estimating the properties of both the liquid and gas are presented. The necessity for utilising such EoSs in bubble collapse simulations is illustrated by the unphysical (spurious) liquid temperature jump formed in the vicinity of the bubble-air interface when simplified EoSs are used. The solved fluid flow equations follow the mechanical equilibrium multiphase method of Kapila. The solver is coded in the AMReX platform, enabling high-performance computation with parallel processing and Adaptive Mesh Refinement for speeding up simulations. It is initially demonstrated that the frequently used Stiffened Gas (SG) EoS overpredicts the liquid temperature at high compression. More sophisticated EoS models, such as the International Association for the Properties of Water and Steam (IAPWS), the Modified Noble Abel Stiffened Gas (MNASG) and a modified Tait EoS introduced here, are also implemented into the flow solver and their differences are highlighted for bubble collapse cases for the first time. Subsequently, application of the developed model to cases of practical interest is showcased. More specifically, simulations of bubble collapse near a solid wall are presented for conditions simulating shock wave lithotripsy (SWL). It is concluded that for such cases, a maximum increase of 25 K of the liquid temperature in contact along the solid wall is caused during the collapse of the air bubble due to shock wave focusing effects. It is also highlighted that the maximum liquid heating varies depending on the initial bubble-wall stand-off distance.

## Highlights

• The lack of accuracy of a commonly used liquid EoS is highlighted through a compression test and spherical bubble collapse.
• Best model predictions are realised when the Modified Tait and MNASG EoS are employed while the latter is more robust at higher compressions.
• Collapse of a bubble near a rigid wall affected by a lithotripter pulse is simulated with a focus on variation of the liquid temperature.
• The effect of the stand-off distance on the liquid temperature along the wall is predicted, showing a significant increase of 25 K, which for cases of lithotripsy can cause thermal damage.

## Graphical abstract

**Figure:**
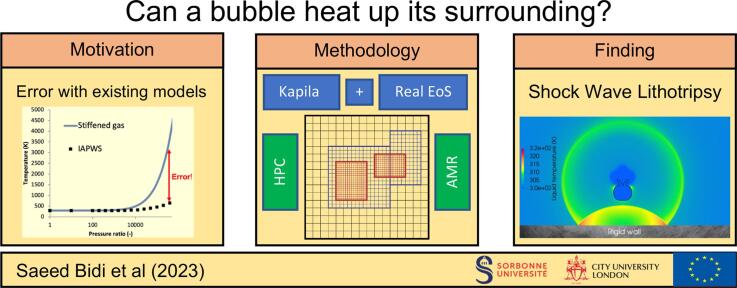

## 1 Introduction

The bulk liquid temperature does not change significantly compared to that of the bubble content [2], [3], [4], [5]. As a result, in the context of thermal effects, attention of the literature is usually directed towards the exceedingly elevated temperature of the contents within the inner bubble, leading to the dissociation of air molecules and triggering chemical reactions. While the surrounding liquid thermodynamics and its impact on the collapse phenomena has not been yet clearly described, the liquid temperature plays a significant role in many applications. Moreover, the liquid thermodynamics determines in high temperature liquids showing an upward trend for both the lifespan and energy of the cavitation bubble [6]. This, however, applies up to a point; beyond that cavitation collapse effects seem to diminish. It is known that the initial liquid temperature affects the bubble dynamics and its lifetime [7], [8], [9] as well as the bubble shape stability [10]. The speed and characteristics of cavitation bubble collapses are also influenced by this factor [11]. Liquid thermodynamics sets the kinetic boundary conditions at the interface which can be used to simulate the phase change [12]. It also influences the formation of cavitation-induced pits [13], ultrasonic nanobubbles generation [14], the critical Weber number for forming surface bubbles [15]. Furthermore, modifying the liquid temperature can control the consequences of the bubble collapse. Research indicates that an increased liquid temperature leads to a reduction in the sonoluminescence intensity [16] by reducing the maximum bubble collapse temperature [17]. Another relevant study [18] confirmed this with a different perspective. They stated that liquid can dissolve more gas at lower temperatures. In addition, it was revealed that when water temperatures are lower, the viscosity of the water rises, leading to enhanced stability of the bubble. Consequently, this results in a more intense collapse and amplified sonoluminescence [6]. There are other studies on the impact of the liquid temperature on the sonoluminescence intensities [19], [20].

The role of the liquid temperature in cavitation has also been investigated in the industry and more practical applications. For example, experiments show that the cavitation damage to the material is affected by liquid temperature by varying the liquid-jet impact force [21]. A relevant experimental study [22] indicates that mass loss and material removal mechanisms are directly influenced by the liquid temperature in vibratory ultrasonic machining. As a different instance, the primary mechanisms underlying High-Intensity Focused Ultrasound (HIFU) devices involves using cavitation and thermal effects to accurately focus acoustic energy in the body's targeted regions. The main idea is to elevate the temperature above a coagulation threshold rapidly, within a few seconds [23], [24], [25], [26], [27] followed by tissue ablation, coagulation, and necrosis through which the tissue will be thermally destroyed while the acoustic energy near the transducer is weak. Applying the HIFU to the targeted area should be carefully controlled in order to keep the rest of the local tissue and skin safe to the greatest extent possible from the thermal and cavitation damage. There are experimental studies on HIFU exposed to tissue-mimicking phantoms or tissues where the temperature increase is measured [28], [29]. Part of this temperature rise can be due to ultrasound absorption and conversion to heat while the other part is due to cavitation [6]. Coupling real thermodynamics through applying accurate equations of state with the CFD simulations will aid in modelling the cavitation-enhanced heating which is vital to ensuring safety and analyse the efficiency of the HIFU treatments [28]. As a different application, the role of liquid temperature in erosion is reviewed in [30]. It is argued that the erosion rate initially escalates with rising liquid temperature until a certain threshold, which is between the freezing and boiling points, after which it exhibits a contrary effect. This has been further confirmed by other researchers [11], [31], [32] where the same temperature dependency was observed for the cavitation impact force.

It should be noted that the surrounding liquid temperature does not remain constant at different collapse stages depending on the settings. For example, it increases during the course of a given HIFU treatment [33]. However, measurement of the temperatures developing during bubble collapse are compromised by the limited spatiotemporal resolution and dynamic range of relevant experimental devices [34]. There have been numerical studies on cavitation where the focus is on the liquid temperature. In some of them, zero-dimensional models have been used to describe the bubble dynamics. In [35], the Keller-Miksis model is used to analyse the spherical bubble collapse energy under ultrasound irradiation. It is demonstrated that the bubble energy slightly increased with the increase in liquid temperature in the range of 283– 333 K. In [7], the role of the liquid temperature in bubble dynamics and lifetime was examined by investigating the dependency of the bubble dynamics on the ambient temperature experimentally and comparing the results with the prediction by the modified Rayleigh Plesset model. Shen et al. [36] developed a bubble dynamics model and calculated the liquid temperature at the bubble wall considering the continuity of the energy flux. The liquid temperature at the exterior of the bubble wall was reported up to 1760 K in the case of a strong collapse confirmed by experiments [37]. Peng et al. [38] investigated the influence of liquid temperature on cavitation collapse intensity using the Tait equation of state for water within a zero-dimensional bubble collapse model. They presented a distribution map of the optimum temperature corresponding to the maximum collapse intensity. Shen et al. [39] calculated the spatial distribution of the liquid temperature near a cavitation bubble wall by adopting a bubble dynamics model. They found that the bubble wall preserves the ambient temperature except at strong collapse where heating raises the temperature up to 1510 K.

As zero-dimensional bubble dynamics models cannot simulate non-spherical bubble collapses, CFD methods have been used to model the liquid thermal effects; in the vast majority of relevant studies, the liquid is deemed incompressible and further the Ideal Gas (IG) EoS is utilised. For example, Fursenko et al. [40] simulated a vapor bubble collapse near the microfiber immersed in a subcooled liquid using the Volume of Fluid (VoF) method. Their results indicate that the average jet velocity decreases significantly with an increase of liquid temperature by 50 K. Yu et al. [41] employed an incompressible VoF method coupled with an ideal equation of state to study the thermodynamic effect during bubble collapse near a rigid boundary. In this study, the effects of the formed jet as well as the initial stand-off distance on the bubble thermodynamics are highlighted. In [42], the VoF method was used coupled with the IG EoS to study the collapse of a compressible gaseous bubble surrounded by incompressible water near a heated wall. The findings indicate that an increase in the initial liquid temperature results in the collapse with lower intensity due to a higher vapour pressure inside the bubble. Popinet et al. [43] explored the viscosity's impact on bubble collapse near solid surfaces where reduction of the jet impact velocity at higher viscosity was observed. There are also some relevant studies for cavitation modelling using mass transfer rates [44], [45], [46], [47], [48], cavitation modelling using air-vapor–liquid and barotropic EoS [49], [50], [51], [52], [53], and heating effects and real-fluid thermodynamic closure in cavitating flows [54], [55], [56].

However, the dynamics of bubble rebound are affected by the incompressibility assumption [57], since an important mechanism of energy dissipation as acoustic energy is ignored. Furthermore, since density changes are inherently ignored, an incompressible assumption cannot describe the relevant thermodynamic effects of compression heating [58]. To account for these deficiencies, further CFD studies have progressively been incorporating compressibility effects in bubble collapse simulations. In the majority of them, the Stiffened Gas (SG) EoS [59], [60], [61], [62], [63], [64] and Tait [65], [66], [67], [68], [69], [70], [71] EoSs have been employed. Although these two EoSs consider liquid compressibility in a straightforward manner, they are unable to describe the liquid thermodynamic behaviour appropriately as the first one uses an unphysical specific heat ratio while the latter does not account for density changes due to temperature variations as explained in Section 2. Therefore, thermal effects were not discussed in these studies. The work of Beig et al. [34] is the most relevant to the present investigation; in this study, a vapour bubble collapse near a solid wall is simulated to quantify the temperature along the wall using the NASG EoS.

The above review shows that simplified thermodynamic assumptions that have been utilised in the vast majority of numerical studies, mainly due to the complexities associated with advanced EoS. It also should be noted that the various EoSs exhibit different accuracy in modelling thermal effects. For instance, in [72] it has been shown that the Gilmore equation for modelling the bubble collapse is used and concluded that the NASG EoS provides a more accurate liquid thermodynamic description than the Tait EoS.

Diffuse Interface Methods (DIM) are widely used for CFD simulation of bubbly flows [73], [74], [75], [76] besides the VoF methods [77], [78], [79], [80]. Their design aims to calculate flow variables within numerically diffused regions adjacent to interfaces [76]. In the present study, we compare for the first time the predictive capability of a variety of liquid EoSs implemented in the model of Kapila [1] which utilises a DIM. Initially the error introduced by SG EoS is highlighted through a compariso with the International Association for the Properties of Water and Steam (IAPWS) database [82] and also against a newly introduced modified Tait EoS. The lack of accuracy of the SG EoS is plainly presented for a spherical bubble collapse case through a comparison with the results obtained with the Modified Noble Abel Stiffened Gas (MNASG) [83]. Following that, 2D axisymmetric simulations of bubble collapse placed at different stand-off distances from a nearby rigid wall and excited by an ultrasound pressure pulse are performed. In these cases, the MNASG EoS is adopted for approximating the liquid phase properties. It is noted that the real gas thermodynamic model of Redlich-Kwong Peng-Robinson (RKPR) [84], as presented in our earlier work [85], is utilised for approximating the dependence of air bubble content of pressure and temperature. As a result, the liquid temperature elevation along the wall is predicted during the collapse or air bubble placed at different stand-off distances from the wall; such predictions represent one of the main novelties of this research.

The rest of this paper is organised as follows: the various liquid EoSs utilised are compared and their differences are highlighted in Section 2. Following that, in Section 3, the numerical method is presented along with a short description of the AMReX platform [86] where the solver is developed. Subsequently, the results are demonstrated in Section 4 where the thermal effects in bubble collapse is the focus. Lastly, the concluding remarks are mentioned.

## 2 Equations of state

The thermodynamic assumptions that affect the materials involved in bubble dynamic cases can play a detrimental role in the temperature distributions and even the dynamics of bubble collapse, although to a much lesser extent. The EoS can play a detrimental role in capturing the aforementioned variations of density during bubble collapse and can shed light on dissipation mechanisms. Further, the formulation of the EoS has a more intrinsic role, as it can affect the predictions of compression heating of the liquid, and thus, consequently influencing the temperature distribution and the heat transfer (from gas to liquid, or from the liquid to nearby solid/soft walls). In fact, in the former work of the authors [85], the relevant EoS has been illustrated to greatly affect compression heating of the bubble contents during strong bubble collapses with the initial pressure ratio (defined as the ratio of external pressure to internal pressure t=0 s) of 353, leading to differences of 4,000 K (or nearly 70 %), between the commonly used IG EoS and real-fluid models. Similar effects, even though to a lesser extent can manifest in the liquid, as it will be further highlighted here. Demonstrating an excellent accuracy and wide range of applicability in our previous work [85], the RKPR EoS is employed in this study for the gas phase in tabulated format with bilinear interpolation as a time-efficient numerical implementation compared to on-the-fly utilisation of the parametric form [87].

### 2.1 Tait EoS

This is a polytropic-type EoS [88], [89] which originally relates liquid pressure to density. The original form of Tait EoS reads as:

(1) $$p=\frac{\rho_{0}c_{0}^{2}}{n}+\left({\left(\frac{\rho }{\rho_{0}}\right)}^{n}-1\right)+p_{0},$$

where p,ρ, and c are pressure, density and speed of sound, respectively. Subscript 0 denotes the reference state. Moreover, exponent n is set to 7.15 for weakly compressible liquids such as water [90]. Although it is rather simple and accurate in predicting liquid densities, it fails to describe density variations due to temperature and, therefore, compression heating. This is indeed the case for all EoS that links density to pressure only, i.e., having the form p=f(ρ). This observation stems from the fact that, in general, entropy can be written as [91]:

(2) $$\mathrm{ds}=\frac{c_{p}}{T}dT+\frac{1}{\rho^{\text{2}}}{\left(\frac{\partial \rho }{\partial T}\right)}_{p}dp.$$

The implication of this equation is that for any EoS written in the form of p=f(ρ), the partial derivative ${\left(\frac{\partial \rho }{\partial T}\right)}_{p}$ is zero, hence entropy changes are a function of temperature only. It becomes thus apparent that in such cases, no matter how much a liquid will be compressed in a reversible and adiabatic manner (i.e., isentropic) it will not heat up, and thus the relevant dissipation effects are ignored. The Tait equation of state has been used in improved extensions of the Rayleigh-Plesset equation, such as Keller-Miksis [92] or Gilmore models [72], or resolved 2D/3D bubble dynamic cases [93], [94], [95]. Alternative forms that incorporate temperature-related effects have been proposed in the past as discussed in [96], see for example Koop [97] or Saurel [98]; however, in both cases the authors have used a simplified representation for internal energy without considering density variation effects.

While common liquids exhibit weak compressibility, the intense conditions during bubble collapse can lead to significant compressions, reaching magnitudes of many GPa. Furthermore, since in reality the liquid density is affected by temperature also, the aforementioned compression will produce heating of the liquid. On the other hand, the simple liquid EoSs that consider density variations of density due to temperature can greatly overestimate this heating. A particular example of such a model, commonly used in bubble dynamics [99], [100], is the SG EoS (for which is the interested reader can refer to the work of Flatten et al. [101] for fundamental thermodynamic relations). To illustrate the deviations that such simplified models can produce, the performance of the simplified and advanced EoSs are compared with the most accurate database IAPWS [82] as a reference formulated in NIST Refprop [102] in the next section.

### 2.2 Modified Tait EoS

Expressing Eq. (1) for density, an alternative proposed hereafter has the following form:

(3) $$\rho ={\left(\frac{p}{\rho_{0}c_{0}^{2}f_{1}\left(T\right)}+1\right)}^{1/n}\rho_{0}f_{2}\left(T\right),$$

where $f_{1}\left(T\right)$ and $f_{2}\left(T\right)$ are functions that need to be determined. Any candidate formula, as the above, can be fitted to IAPWS datasets using NIST Refprop [102]. Two main parameters are required:

1. The speed of sound should vary with respect to temperature and, at high pressures, decrease with increase of temperature. Here a function in the form of $f_{1}\left(T\right)=\sqrt{\left(\frac{a}{T}\right)}$ was chosen.
2. The isobaric density variation with respect to temperature should have an inflexion point, due to the presence of the critical point. Naturally, this can be expressed by using a sigmoid function, such as *tanh*. Hence, a suitable candidate can have the form:

(4) $$\rho \left(p,T\right)={\left(\frac{p}{\rho_{0}c_{0}^{2}\sqrt{aT_{1}/T}}+1\right)}^{1/n}\rho_{0}\left[1-b.\tanh\left(\frac{T-T_{1}}{T_{2}}\right)\right].$$

**Fig. 1:**
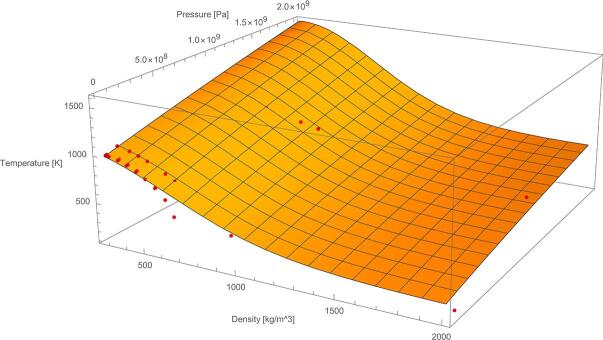
IAPWS data on the surface plot of $\rho \left(p,T\right).$

The selected functions $f_{1}\left(T\right)$ and $f_{2}\left(T\right)$ are produced via data fitting taking numerical stability into account. The incentive behind the general monotonicity is provided in each point, i.e.: (1) speed of sound decreases with respect to temperature and (2) existence of inflection point in density near the critical point. The aforementioned density function defines also the specific volume, $\upsilon =\frac{1}{\rho }.$ Calibration of this formulation is done using IAPWS database, for a range of 280-2000 K and $1000-10^{9}$ Pa. After calibration, the following values for the coefficients are obtained: $T_{1}=650$ Κ, $T_{2}=550$ Κ, a=0.277096868, b=0.659026, $\rho_{0}=708.9997\;$ $\frac{\mathrm{kg}}{m^{3}}$ and n=2. In Fig. 1, red points correspond to IAPWS database, whereas the surface is the plot of the fitted ρ(p,T) function.

Apart from density relation to pressure and temperature, thermodynamic relations for the enthalpy, entropy, speed of sound need to be defined. For heat capacity at constant pressure there are constraints to be satisfied for the EoS to be consistent. In particular, from the definition of enthalpy $\mathrm{dh}=c_{p}dT+\left(\nu -T\frac{d\nu }{\mathrm{dT}}\right)dp$, it has to be an exact differential. Hence, $c_{p}$ and specific volume are linked, as follows:

(5) $$\frac{dc_{p}}{\mathrm{dp}}=\frac{d}{\mathrm{dT}}\left(\nu -T\frac{d\nu }{\mathrm{dT}}\right)\Rightarrow c_{p}=\int_{p_{\mathrm{ref}}}^{p}\frac{d}{\mathrm{dT}}\left(\nu -T\frac{d\nu }{\mathrm{dT}}\right)dp+C_{1}\left(T\right)+C_{2}.$$

The integral above comes directly from the choice of EoS; the only degree of freedom for adjusting $c_{p}$ to a reasonable value comes from the functions $C_{1}\left(T\right)$ and $C_{2}$, which can be chosen to match experimental data. After fitting, $C_{2}$ = 10334 $\frac{J}{\mathrm{kg}.K}$ and $C_{1}=-4.889\left(T-300\right).$ Also, the enthalpy function can then be obtained by integrating:

(6) $$\mathrm{dh}=c_{p}dT+\left(\nu -T\frac{d\nu }{\mathrm{dT}}\right)dp.$$

Similarly, entropy can be obtained by integrating:

(7) $$\mathrm{ds}=\frac{c_{p}}{T}dT+\frac{1}{\rho^{2}}\frac{d\rho }{\mathrm{dT}}dp.$$

Further, various derivatives of density and enthalpy can be defined, once the respective formulation is obtained, as $\frac{\mathrm{dh}}{\mathrm{dT}}$, $\frac{\mathrm{dh}}{\mathrm{dp}},$ $\frac{d\rho }{\mathrm{dT}}$, and $\frac{d\rho }{\mathrm{dp}}$, which can be further used to define speed of sound:

(8) $$c=\sqrt{\frac{\rho \frac{\mathrm{dh}}{\mathrm{dT}}}{\rho \frac{d\rho }{\mathrm{dp}}\frac{\mathrm{dh}}{\mathrm{dT}}+\frac{d\rho }{\mathrm{dT}}\left(1-\rho \frac{\mathrm{dh}}{\mathrm{dp}}\right)}},$$

and heat capacity at constant volume, $c_{v}$, as:

(9) $$c_{v}=c_{p}-\frac{{\left(\frac{1}{v}\frac{\mathrm{dv}}{\mathrm{dT}}\right)}^{2}}{\left(\frac{1}{\rho }\frac{d\rho }{\mathrm{dp}}\right)\rho }.$$

### 2.3 Noble Abel Stiffened gas (NASG) and MNASG EoSs

The SG EoS involves molecular attractive and agitative forces. The aim of the Noble Abel Stiffened Gas (NASG) is to add repulsive forces to the SG EoS in order to reduce the density error as shown in [103]:

(10) $$p=\left(\gamma -1\right)\frac{\left(e-q\right)}{\left(\upsilon -b\right)}-\gamma p_{\infty },$$

where q, $p_{\infty }$, and b are the fluid heat bond, a characteristic constant, and co-volume as the volume of the molecules’ pack, respectively, all depending on the medium. More specifically, the molecular agitation is included in the term $\left(\gamma -1\right)\left(e-q\right)$ while the repulsive forces are represented by $\left(\upsilon -b\right)$. The term $\gamma p_{\infty }$ represents the attractive effects leading to matter cohesion in liquid and solid states. It is noted that setting q=b=0 recovers the original SG EoS. The temperature-based representation of the NASG EoS is derived from the Maxwell rules [103]:

(11) $$T=\frac{\left(\upsilon -b\right)\left(p+p_{\infty }\right)}{\left(\gamma -1\right)c_{v}},$$

in which $c_{v}$ is heat capacity at constant volume. Also, the speed of sound in NASG is obtained from:

(12) $$c^{2}=\frac{\gamma \upsilon^{2}\left(p+p_{\infty }\right)}{\left(\upsilon -b\right)}.$$

The NASG coefficients for liquid water are presented in Table 1.

**Table 1:** NASG coefficients for liquid water.

$c_{v}\left(\frac{J}{kgK}\right)$	γ	$p_{\infty }\left(Pa\right)$	$b\left(\frac{m^{3}}{kg}\right)$	$q\left(\frac{J}{kg}\right)$
3610	1.19	$7028\times 10^{5}$	$6.61\times 10^{-4}$	-1177788

In [83], it was shown that the NASG EoS overpredicts the water density at low pressures when using the saturation value as the reference state. Therefore, the reference states of the variables were modified to generate new values for $p_{\infty }$ and b as indicated in Table 2. The resulted thermodynamic closure is the MNASG EoS.

**Table 2:** MNASG coefficients for liquid water.

$c_{v}\left(\frac{J}{kgK}\right)$	γ	$p_{\infty }\left(Pa\right)$	$b\left(\frac{m^{3}}{kg}\right)$	$q\left(\frac{J}{kg}\right)$
3610	1.19	$6217.8\times 10^{5}$	$6.7212\times 10^{-4}$	-1177788

It is noted that a set of γ=4, $p_{\infty }=6\times 10^{8}$, and b=q=0 converts the above formulae to the common SG EoS for water.

### 2.4 Comparison of the liquid models

**Fig. 2:**
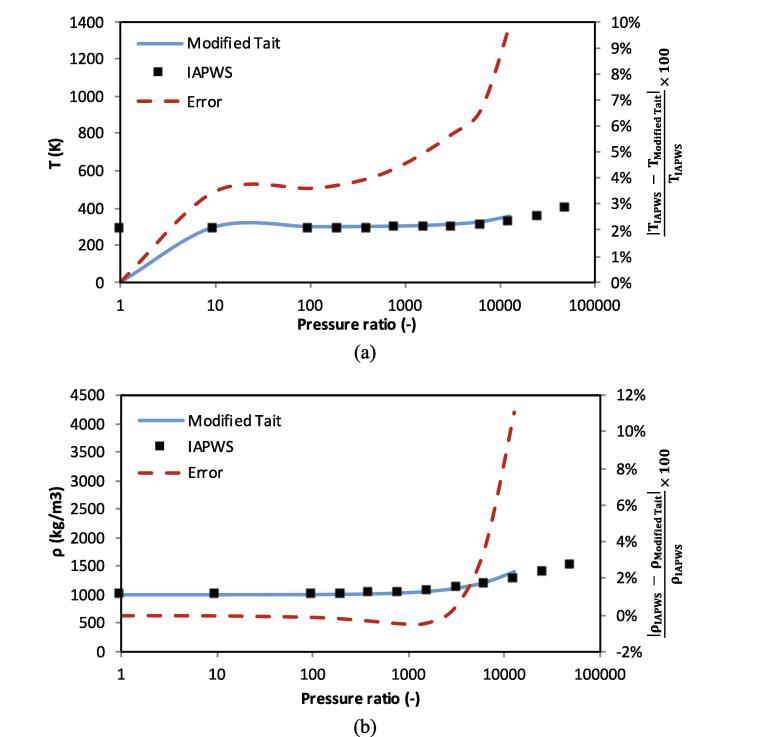
Comparison of temperature (a) and density (b) obtained with the modified Tait EoS and the IAPWS data at different compression ratios. Squares represent the IAPWS reference, the blue line results of the Modified Tait EoS and the red line is the error in percentage corresponding to the right axis. (For interpretation of the references to colour in this figure legend, the reader is referred to the web version of this article.)

**Fig. 3:**
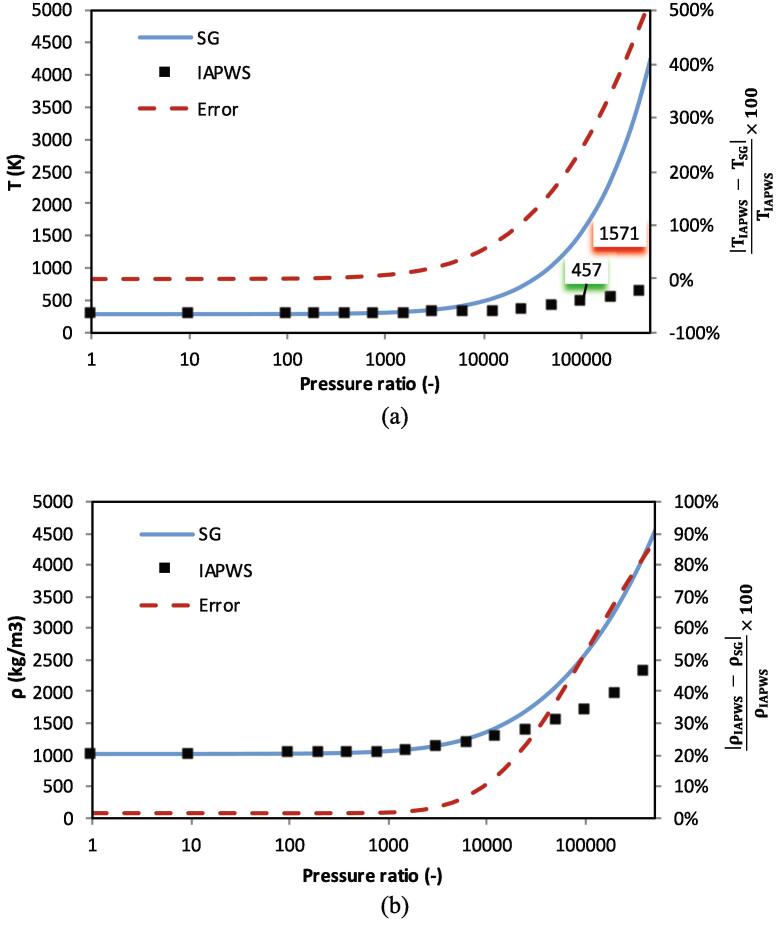
Comparison of temperature (a) and density (b) obtained with the SG EoS commonly used in bubble dynamic studies and the IAPWS data at different compression ratios. Squares represent the IAPWS reference, the blue line results of the SG EoS and the red line is the error in percentage corresponding to the right axis. (For interpretation of the references to colour in this figure legend, the reader is referred to the web version of this article.)

To demonstrate the applicability of the modified Tait EoS both in terms of temperature and density prediction, isentropic compression of liquid water is examined in Fig. 2. The compression starts from 1 bar and 288.15 K. After compression at a given pressure ratio, the water density and temperature increase. As shown below, in the range of calibration the accuracy is rather decent, below 10%, both in terms of temperature and density prediction. In Fig. 3, a similar test is performed with the SG EoS with the same initialisation. As shown, the SG EoS tends to dramatically over-predict the resulting compression heating at high pressures. At a pressure ratio of $10^{5}$, i.e., when liquid is compressed to 10 GPa, the SG EoS predicts a temperature increase of roughly 1282 K. Contrary to this prediction, the IAPWS data predicts a temperature rise of roughly 169 K, almost an order of magnitude lower that the SG prediction. Similarly, a significant error in density prediction of ≈50% with the SG EoS is observed at this compression ratio.

It becomes apparent that the above can play an important role in research on heat transfer of collapsing bubbles; over/under-prediction of liquid temperature due to the adopted EoS can affect the heat fluxes to or from the bubble. Furthermore, the overestimation of compression heating can be observed even at passing shock waves, emitted during bubble collapses as will be shown in the results section.

Advanced models such as IAPWS come with a larger complexity, which makes them rather cumbersome to implement. Whereas tabulation methods can be applied (see [85], [104]) to expedite calculations, their inherent ability to capture phase transitions can cause problems with the numerical solution of the flow equations. Hence, it is of interest to devise robust and versatile thermodynamic closures for liquids, suited for studying bubble collapses.

In the present study, the aforementioned thermodynamic closures, i.e., IAPWS, modified Tait, MNASG, and SG for liquid and RKPR for gas are implemented on a multiphase DIM known as Kapila model outlined in the subsequent section. While MNASG and SG EoSs are applied in their parametric forms, the IAPWS data, modified Tait, and RKPR EoSs are implemented through tabulated format first because of the intricate nature associated with implementation and secondly for faster computation [85]. Each table features a grid with fixed intervals of T and $\log_{10}p$ set in a rectangular layout. The temperature and pressure range span $\left[274,3000\right]$ K and $\left[2300,10^{9.3}\right]$ Pa with 2181 and 745 cells in each direction, respectively, for the IAPWS and modified Tait EoSs. The pressure range is wide enough for low to medium bubble collapse cases up to pressure ratio of ≈175. For more intense collapses, the MNASG is applied. For the gas phase, the RKPR EoS is applied the temperature and pressure ranges of which are $\left[60,17000\right]$ K and $\left[2300,{1.1\times 10}^{10}\right]$ Pa with 121 and 375 cells in each direction, respectively.

## 3 Numerical model and methodology

There are many multiphase methods developed for compressible flows in the literature. Those with the least restrictive assumptions consider that the phases are in complete disequilibrium [105], [106] indicating that every phase possesses its own velocity, pressure, and temperature. However, due to their complexity, equilibrium assumptions between the co-flowing phases are often considered. In our previous works [85], we expanded the model of [107] which assumes velocity equilibrium but considers pressure and temperature disequilibrium to incorporate tabulated EoSs. There are further models in this context with a variety of equilibrium assumptions [1], [75], [108], [109]. In the present study, the bubble collapse is modelled using a 5-equation mechanical equilibrium multiphase model of Kapila [1], which stems from the full disequilibrium model of Baer-Nunziato [105] assuming zero relaxation time for both velocity and pressure. This model is the most widely used DIM according to [81] for the simulation of compressible two-phase flows. It involves a volume fraction equation, mass balance equations for each phase, a mixture momentum equation, and a total energy equation. Neglecting the effects of viscosity, heat conductivity, surface tension, and phase transition, the model in 1D spherical and 2D axisymmetric coordinates (cylindrical coordinates with azimuthal symmetry) reads:

(13) $$\frac{\partial q}{\partial t}+\frac{\partial F}{\partial r}+\frac{\partial G}{\partial z}=s_{nc}\left(q\right)+s_{g}\left(q\right),$$

where:

$$q=\left[\begin{array}{c} \alpha_{1}\\ \alpha_{1}\rho_{1}\\ \begin{array}{c} \alpha_{2}\rho_{2}\\ \begin{array}{c} \begin{array}{c} \rho u\\ \rho w \end{array}\\ \begin{array}{c} \rho E \end{array} \end{array} \end{array} \end{array}\right],F=\left[\begin{array}{c} \alpha_{1}u\\ \alpha_{1}\rho_{1}u\\ \begin{array}{c} \alpha_{2}\rho_{2}u\\ \begin{array}{c} \begin{array}{c} \rho u^{2}+p\\ \rho uw \end{array}\\ \begin{array}{c} \left(\rho E+p\right)u \end{array} \end{array} \end{array} \end{array}\right],G=\left[\begin{array}{c} \alpha_{1}w\\ \alpha_{1}\rho_{1}w\\ \begin{array}{c} \alpha_{2}\rho_{2}w\\ \begin{array}{c} \begin{array}{c} \rho uw\\ \rho w^{2}+p \end{array}\\ \begin{array}{c} \left(\rho E+p\right)w \end{array} \end{array} \end{array} \end{array}\right],$$

$$\begin{array}{c} s_{nc}=\left[\begin{array}{c} \left(K+\alpha_{1}\right)\left(\frac{\partial u}{\partial r}+\frac{\partial w}{\partial z}\right)\\ 0\\ \begin{array}{c} 0\\ \begin{array}{c} 0\\ \begin{array}{c} \begin{array}{c} 0\\ 0 \end{array} \end{array} \end{array} \end{array} \end{array}\right],\\ s_{g}=-\frac{\beta }{r}\left[\begin{array}{c} -Ku\\ \alpha_{1}\rho_{1}u\\ \begin{array}{c} \alpha_{2}\rho_{2}u\\ \begin{array}{c} \rho u^{2}\\ \begin{array}{c} \begin{array}{c} \rho uw\\ u\left(\rho E+p\right) \end{array} \end{array} \end{array} \end{array} \end{array}\right], \end{array}$$

where value of 2 and 1 for the coordinates switching parameter β correspond to the 1D spherical the r-direction and 2D axisymmetric coordinates in the (r,z) directions, respectively. Also, the following notation is adopted: t (time), ρ (density), p (pressure), α(volume fraction), u (r-direction velocity), w (z-direction velocity), E(specific total energy). Moreover, K is defined as:

(14) $$K=\frac{\rho_{2}c_{2}^{2}-\rho_{1}c_{1}^{2}}{\frac{\rho_{2}c_{2}^{2}}{\alpha_{2}}+\frac{\rho_{1}c_{1}^{2}}{\alpha_{1}}},$$

which represents the decrease and increase of the gas volume fraction in the cases of compression and rarefaction waves, respectively. Also, $c_{k}$ is the speed of sound for phase k. The mixture speed of sound c follows Woods relation [110] as follows:

(15) $$\frac{1}{\rho c^{2}}=\frac{\alpha_{1}}{\rho_{1}c_{1}^{2}}+\frac{\alpha_{2}}{\rho_{2}c_{2}^{2}}.$$

As the system of Eq. (13) shows, this model assumes immediate velocity and pressure equilibrium between the phases. The system is solved using a finite volume Godunov method [111] with the second-order MUSCL scheme [112] to reconstruct the primitive variables at the cell boundary. Also, the HLLC approximate solver [113] is employed for solving the Riemann problem at each cell boundary. The traditional Godunov scheme for updating the conservative component of the system is as follows:

(16) $$U_{i,j}^{n+1}=U_{i,j}^{n}-\frac{\Delta t}{\Delta r}\left(F_{cons}^{*}\left(U_{i,j}^{n},U_{i+1,j}^{n}\right)-F_{cons}^{*}\left(U_{i-1,j}^{n},U_{i,j}^{n}\right)\right)-\frac{\Delta t}{\Delta z}\left(G_{cons}^{*}\left(U_{i,j}^{n},U_{i,j+1}^{n}\right)-G_{cons}^{*}\left(U_{i,j-1}^{n},U_{i,j}^{n}\right)\right)+\Delta ts_{g,cons},$$

in which:

$$U={\left[\begin{array}{c} \alpha_{1}\rho_{1}\\ \alpha_{2}\rho_{2}\\ \begin{array}{c} \rho u\\ \begin{array}{c} \rho w\\ \rho E \end{array} \end{array} \end{array}\right]}^{T},F_{cons}={\left[\begin{array}{c} \alpha_{1}\rho_{1}u\\ \alpha_{2}\rho_{2}u\\ \begin{array}{c} \rho u^{2}+p\\ \begin{array}{c} \rho uw\\ \left(\rho E+p\right)u \end{array} \end{array} \end{array}\right]}^{T},G_{cons}={\left[\begin{array}{c} \alpha_{1}\rho_{1}w\\ \alpha_{2}\rho_{2}w\\ \begin{array}{c} \rho uw\\ \begin{array}{c} \rho w^{2}+p\\ \left(\rho E+p\right)w \end{array} \end{array} \end{array}\right]}^{T},s_{g,cons}=-\frac{\beta }{r}{\left[\begin{array}{c} \alpha_{1}\rho_{1}u\\ \alpha_{2}\rho_{2}u\\ \begin{array}{c} \rho u^{2}\\ \begin{array}{c} \rho uw\\ \begin{array}{c} u\left(\rho E+p\right) \end{array} \end{array} \end{array} \end{array}\right]}^{T}.$$

The finite volume cell index in (r,z) direction is represented by (i,j) while superscript n indicates the time step. The perturbated state is represented by superscript’∗’. Calculation of $F_{cons}^{*}$ and $G_{cons}^{*}$ using the HLLC Riemann solver is discussed in [107]. The volume integral is approximated using a midpoint rule and the divergences with a centered scheme [107] for the non-conservative component:

(17) $$\alpha_{i,j}^{n+1}=\alpha_{i,j}^{n}-\frac{\Delta t}{\Delta r}\left({\left(u\alpha \right)}_{i+\frac{1}{2},j}^{*}-{\left(u\alpha \right)}_{i-\frac{1}{2},j}^{*}-\left(\alpha_{i,j}^{n}+K\right)\left(u_{i+\frac{1}{2},j}^{*}-u_{i-\frac{1}{2},j}^{*}\right)\right)-\frac{\Delta t}{\Delta z}\left({\left(w\alpha \right)}_{i,j+\frac{1}{2}}^{*}-{\left(w\alpha \right)}_{i,j-\frac{1}{2}}^{*}-\left(\alpha_{i,j}^{n}+K\right)\left(w_{i,j+\frac{1}{2}}^{*}-w_{i,j-\frac{1}{2}}^{*}\right)\right)+\Delta t\frac{\beta Ku}{r}.$$

At each stage, the mixture pressure is computed using the mixture rule for the internal energy e through the following steps:

(18) $$e=Y_{1}e_{1}+Y_{2}e_{2},$$

where $Y_{k}=\frac{\alpha_{k}\rho_{k}}{\rho }$ is the mass fraction for phase k and the phasic internal energies $e_{k}$ are inserted as functions of pressure and temperature $e_{k}=e_{k}\left(p,T_{k}\right)$ through either tabulated data or the analytical relations in the case of the parametric equations of state. The procedure is summarized as follows:

The mixture internal energy e is calculated from the total and kinetic energies:
  (19) $$e=E-\frac{1}{2}\left(u^{2}+w^{2}\right).$$
The mixture rule for the internal energies is considered as:
  (20) $$e=Y_{1}e_{1}+Y_{2}e_{2},$$
  where $Y_{k}=\frac{\alpha_{k}\rho_{k}}{\rho }$ and $e_{k}$ are the mass fraction and phasic internal energy for phase k. In the case of using parametric equations of state for both phases the $e_{1}$ and $e_{2}$ are inserted as functions of pressure and densities $e_{k}=e_{k}\left(p,\rho_{k}\right)$ based on the equations of state such that the pressure will be the only unknown. For instance, in the case of the NASG EoSs, Eq. (20) reads:
  (21) $$p=\frac{\rho e-\left(\frac{\alpha_{1}\left(1-\rho_{1}b_{1}\right)\gamma_{1}p_{\infty ,1}}{\gamma_{1}-1}+\frac{\alpha_{2}{\left(1-\rho_{2}b_{2}\right)\gamma }_{2}p_{\infty ,2}}{\gamma_{2}-1}\right)-\left(\alpha_{1}\rho_{1}q_{1}+\alpha_{2}\rho_{2}q_{2}\right)}{\frac{\alpha_{1}\left(1-\rho_{1}b_{1}\right)}{\gamma_{1}-1}+\frac{\alpha_{2}\left(1-\rho_{2}b_{2}\right)}{\gamma_{2}-1}}.$$
  However, for the more complex equations of state, tabulated or parametric, there is no analytical solution of Eq. (20). Therefore, an iterative method is proposed by defining an error function based on Eq. (19):
  (22) $$∊=e-E+\frac{1}{2}\left(u^{2}+w^{2}\right).$$
  Newton’s method for pressure is used as:
  (23) $$p^{\left(n+1\right)}=p^{n}-{\left(\frac{∊}{{∊}_{p}^{\prime }}\right)}^{n},$$
  where ${∊}_{p}^{\prime }$ is the derivative of the error function with respect to the pressure at constant density estimated as:
  (24) $${∊}_{p}^{\prime }=\frac{\Delta ∊}{\Delta p}=\frac{e\left(p+\Delta p\right)-e\left(p\right)}{\Delta p},$$
  where Δp represents a small change in pressure and can be estimated based on the pressure from the previous loop $\Delta p=\xi_{p}p$ for which $\xi_{p}=10^{-3}$ is recommended. Moreover, the initial guess values are considered based on the previous time step. Also, an under-relaxation treatment is also considered to ensure stability. The pressure value is accepted once the solution converges $\left|\frac{\left(p^{\left(n+1\right)}-p^{n}\right)}{p^{\left(n+1\right)}}\right|<\varepsilon$ with a suggested value of $\varepsilon =10^{-3}$.

### 3.1 AMReX implementation

**Fig. 4:**
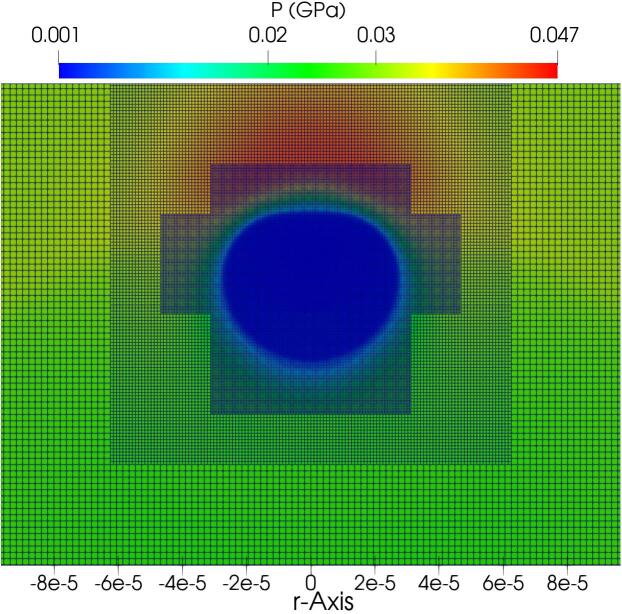
Block-structured grid with 3 levels of refinement.

In the present work, the Kapila model with advanced thermodynamics has been implemented in the open source package AMReX [86]; this offers parallel Adaptive Mesh Refinement (AMR) data structures and linear solvers designed for building massively scalable block-structured AMR applications. The platform provides C++ and Fortran interfaces and supports multidimensional systems with parallelisation via MPI or OpenMP (or hybrid) on High Performance Computing (HPC) architectures. It also contains various tools for solving PDEs on structured grids. The first level grid (the coarsest) is generated at the beginning of the simulation, covering the entire domain. Subsequently, the refined levels are built and dynamically change during the simulation depending on selected refinement criteria. In this context, we employ a density gradient approach, characterised by a minimum gradient threshold denoted as ${\nabla \rho }_{\min}$, to guide the refinement process. Whenever the density gradient between adjacent cells exceeds this specified threshold, an additional grid level is generated. It is important to note that this assessment is performed at intervals of every $n_{\mathrm{AMR}}$ time steps. This criterion plays a pivotal role in our strategy, serving to both enhance the resolution of interfaces, mitigating diffusion, and optimising computational efficiency by utilising coarser grids in regions exhibiting lower density gradients. Our analysis showed that values of ${\nabla \rho }_{\min}=1kg/m^{3}$ and $n_{\mathrm{AMR}}=5$ lead to an efficient computation. To gain insight into the functioning of block-structured Adaptive Mesh Refinement techniques, Fig. 4 illustrates the multilevel refinement approach showcasing three distinct refinement levels at a time step during the non-spherical collapse which will be elaborated in the next section. The AMReX library has been extensively documented in [86], [114] and employed for CFD simulations e.g., see [115], [116], [117].

## 4 Results

In this section, we initially investigate the role of the EoS for a pure water shock tube test case. Following that, a spherical bubble collapse is simulated and the results are compared with our previous work [85] employing the relaxation model. Both bubble hydrodynamics and thermodynamics align perfectly, demonstrating the validity of the implementation of the developed tabulated EoS. Subsequently, the effect of the liquid EoS on the spherical bubble collapse is investigated for different pressure ratios. Lastly, a 2D non-spherical collapse is simulated for which the liquid temperature variation along the wall varying with the initial stand-off distance is reported.

In all simulations, three levels of grid refinement are considered, which lead to the grid-independent results. Moreover, there is a minimum volume fraction $\alpha_{\min}$ of each phase in the entire domain in the initial setups to ensure the hyperbolicity of the system. Moreover, the monotonized central slop limiter is used for the MUSCL reconstruction scheme, as explained in [107]. The time step varies based on the CFL number which is set to 0.5. Moreover, it is assumed that the bubble is solely filled with air; the initial interior pressure $p_{\mathrm{air}}$ is uniform, whereas in the pressure of the water surrounding the bubble follows the distribution described in [118]:

(25) $$p_{\mathrm{water}}\left(r\right)=p_{f}+\frac{R_{0}}{r}\left(p_{\mathrm{air}}-p_{f}\right),$$

where $p_{f}$ denotes the far-field pressure.

### 4.1 1D shock tube

**Fig. 5:**
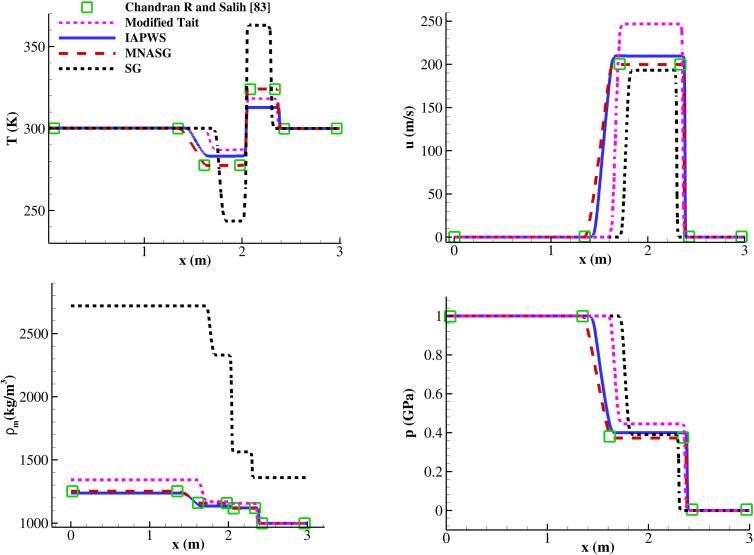
Water shock tube profile after 200μs with various liquid EoSs for water compared with reference [83].

Shock tube problems serve as benchmark tests for the predictive capability of EoSs. Herein, a 3 m long pure water shock tube case has been considered as described in [83]. The diaphragm is placed at x=2 m. Initially, uniform temperature of 300 K prevails while $p=10^{9}$ Pa in x<2 m and $p=10^{5}$ Pa in x≥2 m. Velocity starts at zero throughout the domain. The discretisation accounts for a set number of 1,000 cells, which has been found in prior studies to be sufficient [107]. As discerned from Fig. 5, the SG EoS exhibits a notable temperature jump numerically near the density discontinuity. It is also observed that the results derived from utilising the MNASG EoS align with findings reported in [83]. The SG and the modified Tait EoSs lead to the maximum 16% and minimum 1% temperature overpredictions, respectively, relative to the highly precise IAPWS EoS. This is consistent with the findings as outlined in Section 2. It is further noticed that the developed modified Tait EoS has a limited valid range compared to the MNASG for the bubble collapse cases (initial pressure ratio of 180). This might be enhanced in the future by making more astute choices of functions in Eq. (3).

### 4.2 1D bubble collapse

The purpose of this test case is twofold; firstly, to validate the solver against widely used bubble collapse cases and secondly, to compare the results against those reported in [85]. More specifically, an 1D spherical bubble with $R_{0}=1$ mm is forced to collapse under the influence of a pressure gradient. The case set up is as follows: $p_{\mathrm{air}}=1.01325\times 10^{5}$ Pa, $\rho_{\mathrm{air}}=1.225\frac{\mathrm{kg}}{m^{3}}$, $p_{f}=3.57589\times 10^{7}$ Pa, $\rho_{\mathrm{water}}=998.2\frac{\mathrm{kg}}{m^{3}}$ is set up. The domain length is $L_{0}=20$ mm and it has been discretised using 2,000 uniformly distributed cells. The plotted radius and time are non-dimensionalised with initial radius $R_{0}$ and the Rayleigh collapse time, respectively:

(26) $$R^{*}=\frac{r}{R_{0}},$$

(27) $$t^{*}=\frac{t}{0.915R_{0}\sqrt{\frac{\rho_{\mathrm{water}}}{p_{f}}}}.$$

**Fig. 6:**
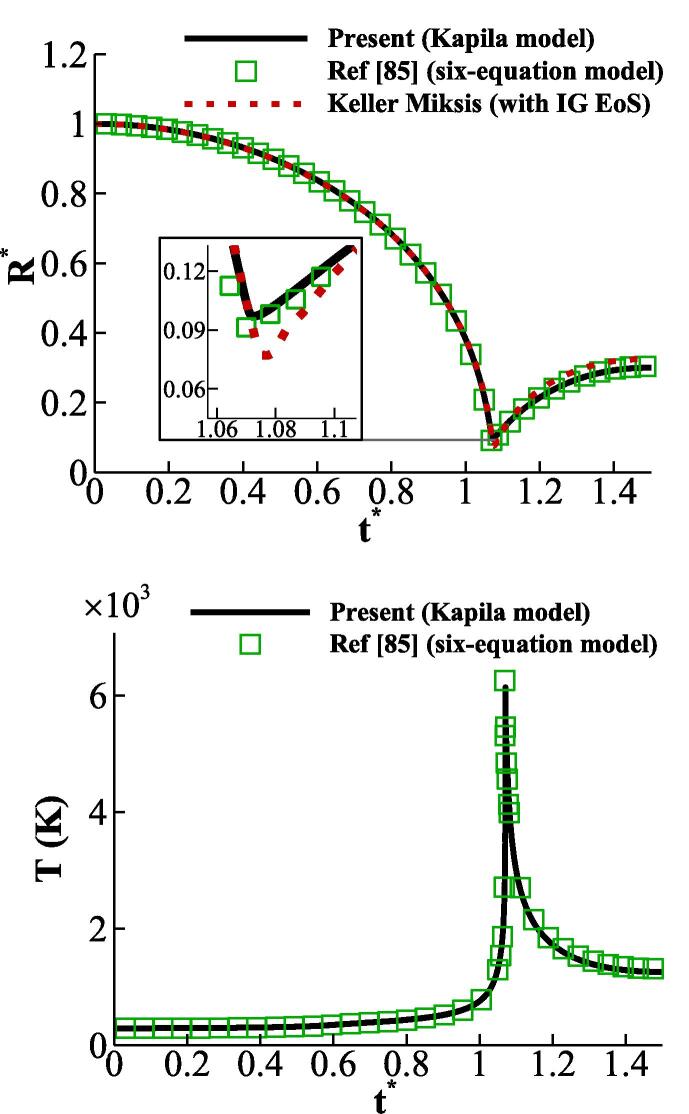
Comparison of the bubble dynamics (a) and temperature (b) obtained with Kapila model in the present study to the ones with the six-equation model in [85].

For comparison purposes, the RKPR and SG EoSs have been selected for the gas and liquid phases, respectively, similarly to our former study [85]. Fig. 6a shows that excellent agreement between the present simulation against the corresponding results obtained with the Keller-Miksis model; in the latter, the IG EoS is used for the gas phase. This agreement is expected since it is known that the gas EoS has a minimal influence on the change of bubble radius during the collapse [85]. Moreover, the collapse dynamics and the space-averaged bubble temperature are identical to those reported in [85]; it is noted that in this study, a different diffused interface model known as the ‘six-equation model’ is used with the same equations of state, i.e., the pair of the RKPR and SG EoSs.

**Fig. 7:**
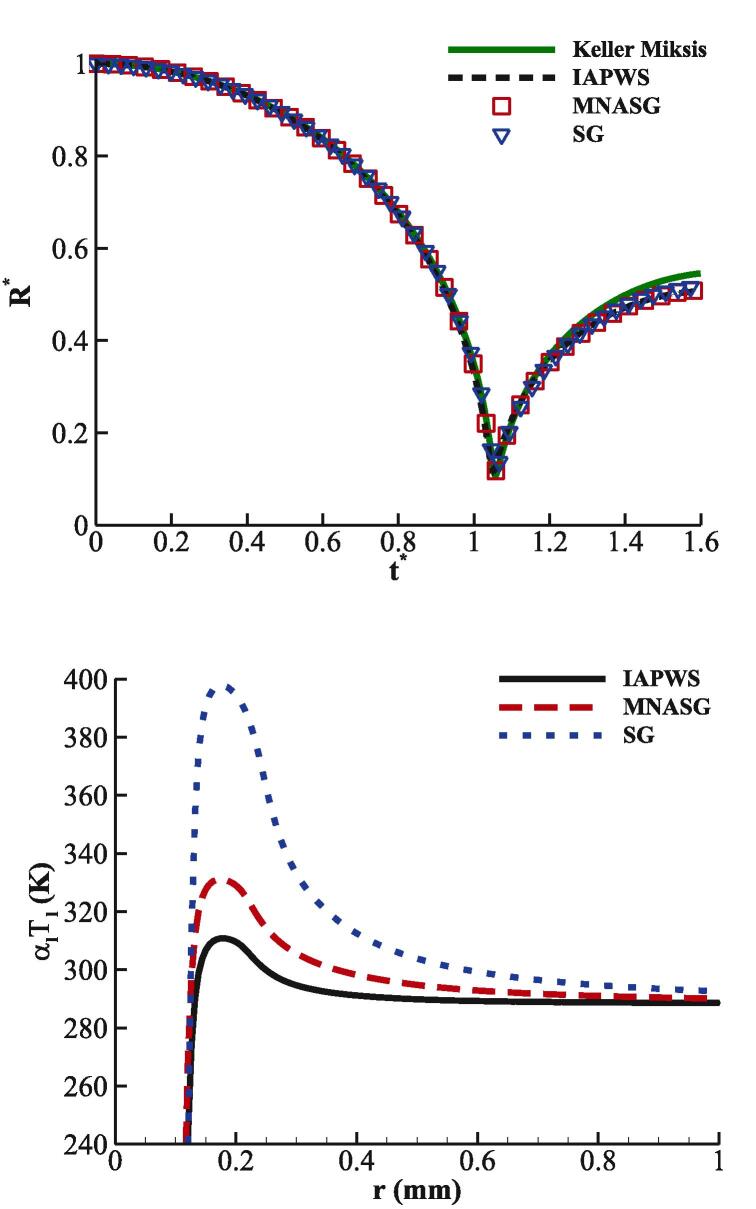
(a) Bubble dynamics and (b) water temperature predicted by the IAPWS and MNASG EoSs.

After presenting the aforementioned benchmark cases, demonstration of the relative errors that can result from the SG EoS will be reported. In this regard, the initialisation of the previous case is slightly modified by replacing the liquid density with its temperature and lowering the initial pressure ratio as shown in Table 3. This simulation is performed with RKPR EoS for the gas and with the SG, IAPWS, and MNASG EoSs for the liquid phase. Predictions for the temperature in the vicinity of the bubble interface are illustrated in Fig. 7b. It is observed that the SG EoS results in up to 30% temperature overprediction compared to the IAPWS EoS, while the maximum overprediction from the MNASG EoS is below 6%. It is noteworthy to mention that the original version of the NASG EoS could not accurately depict the dynamics of the collapse.

**Table 3:** Initial conditions for the collapse case with real thermodynamics.

$p_{\mathrm{air}}$(Pa)	$p_{f}$(Pa)	$\rho_{\mathrm{air}}\left(\frac{\mathrm{kg}}{m^{3}}\right)$	$T_{\mathrm{water}}$(K)
$10^{5}$	${8.7\times 10}^{6}$	1.225	288

**Fig. 8:**
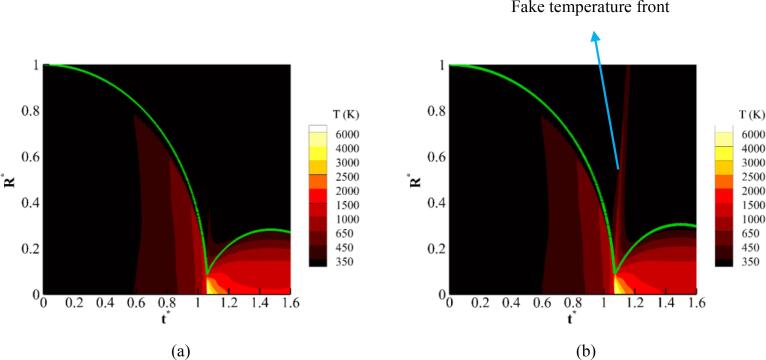
Spatio-temporal change of the mixture temperature with RKPR EoS for air; and a) MNASG and b) SG EoS for water.

Since the IAPWS and the modified Tait fail at higher compressions, a stronger collapse with the initial $p_{f}=3.57589\times 10^{7}$ Pa is simulated here using the MNASG and SG EoSs. The rest of initialisation parameters are the same as in Table 3. It is noted again that the gas EoS is RKPR. The spatio-temporal change of the gas–liquid mixture temperature approximated by ${T=Y}_{g}{T_{g}+Y}_{l}T_{l}$ EoSs is plotted when using MNASG EoS in Fig. 8a and SG EoS in Fig. 8b. The occurrence of a fake temperature front is much more evident when the SG EoS with the maximum of ≈700 K while it is much less ≈450 K when using MNASG EoS. This indicates that the SG EoS overpredicts a travelling heat wave. It can be also observed that as this will temperature decreases both with time after the collapse point and space move towards the far-field.

### 4.3 Axisymmetric collapse

One of the diverse applications of ultrasound cavitation in biomedicine is shock wave lithotripsy (SWL). The mechanism of lithotripsy involves producing short focused microsecond pulses that cause shockwave penetrating the body at a target site. During a treatment session, numerous pulses are administered, typically at frequencies ranging between 1 and 2 Hz. Cavitation is known to affect both the intended fragmentation of stones and the unintended harm to surrounding tissues [119]. While increasing the pulse rates could expedite the treatment process, it could also lead to tissue damage [119], [24].

**Fig. 9:**
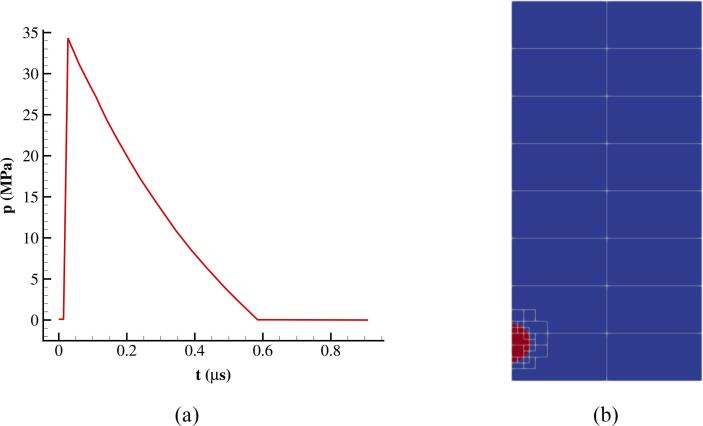
(a) Pressure pulse of the lithotripter and (b) Schematic of the initial setup for the non-spherical collapse case with block structured grid in AMReX.

A simplified representation for SWL can be examined as follows. A compressive shock front from the upper boundary depicted in Fig. 9a represents the lithotripter pulse without the tensile part propagating in time; this is based on an analytical function described in [120] resembling that of an electrohydraulic lithotripter system Dornier HM3, which is a commonly used lithotripter. This test case was first introduced in the work of [120], where they studied the wall pressure subjected to the bubble collapse. In this setup, infinite impedance for the kidney stone is assumed to avoid any wave absorption in the boundary.

Initially, the pressure is atmospheric in the whole computational domain; the water and air densities are $\rho_{\mathrm{water}}=998.2\;kg/m^{3}$ and $\rho_{\mathrm{air}}=1.125\;kg/{\text{m}}^{3}$, respectively. To reduce the computational cost, the case is simulated in 2D axisymmetric coordinates instead of the full 3D configuration. The schematic of the geometry is presented in Fig. 9b where the domain is divided into separate blocks while the grid is refined in the proximity of the liquid–air interface. Reflective boundary condition is used on the axis of symmetry whereas for the right side and the bottom wall, the non-reflective and no-slip boundary conditions have been used, respectively. The bubble has an initial radius of $R_{0}=0.05$ mm while the initial stand-off distance, defined as the minimum distance between the bubble centre and the wall, is ${H_{0}=2R}_{0}$.

**Fig. 10:**
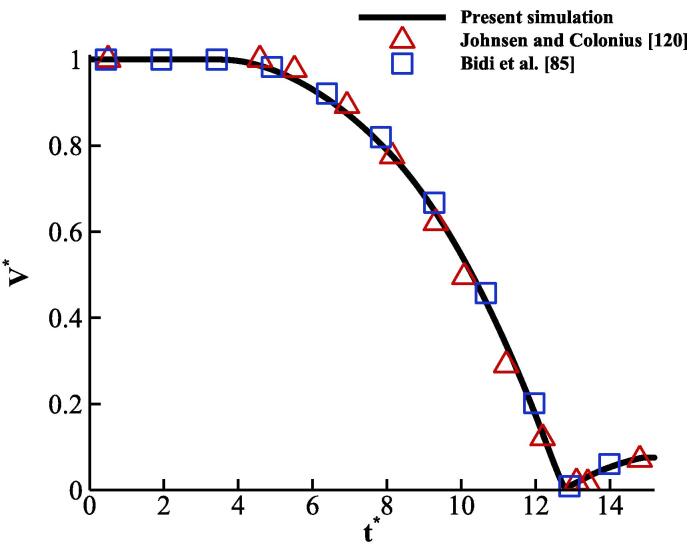
Bubble dynamics of shock-induced collapsing bubble using RKPR and MNASG EoSs compared with [85], [120].

The temporal variation of the bubble volume is initially shown in Fig. 10. For consistency with the results reported in [120], the bubble volume is normalised with its initial value $V^{*}=V/V_{0}$ and the time is non-dimensional using $t^{*}=tc_{L}/R_{0}$. In this case, $c_{L}=1,647\;m/s$ is the reference speed of sound. The results obtained with the ideal and real gas EoSs are compared with the study of [120]; overall, good agreement is achieved, as expected.

**Fig. 11:**
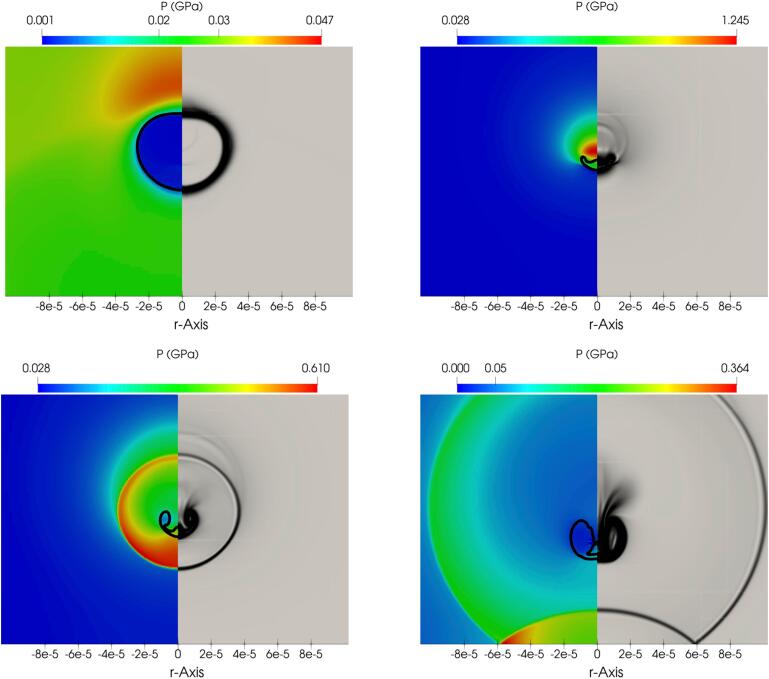
Pressure field (left half), numerical Schlieren (right half) at different collapse stages: a) $t^{*}=11.50$, b) $t^{*}=12.65$, c) $t^{*}=13.07$, and d) $t^{*}=14.41$.

**Fig. 12:**
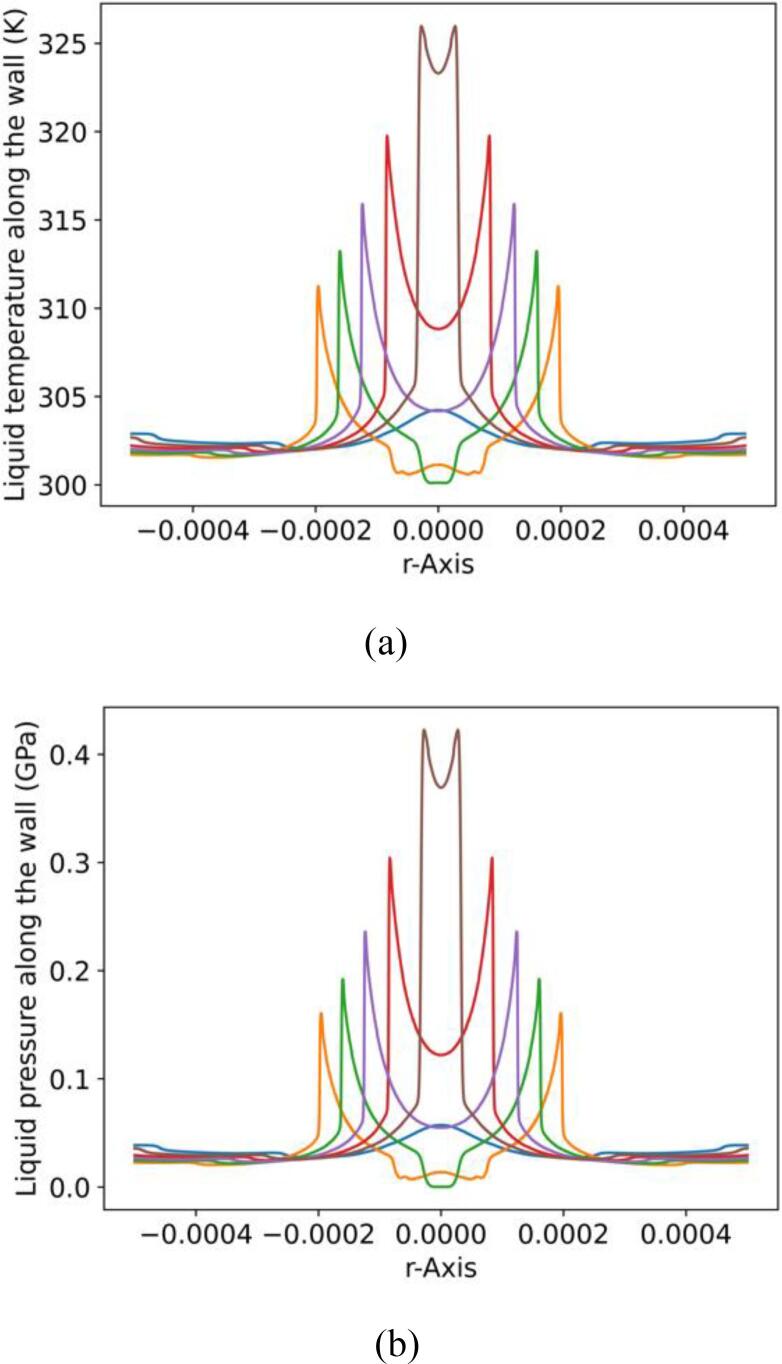
Liquid temperature (a) and pressure (b) along the wall at different times after the shock hits the wall.

Further details of the collapse process are illustrated in the following plots. More specifically, the relevant pressure and numerical Schlieren contours from various moments during the collapse are showcased in Fig. 11. For these simulations, the MNASG has been utilised as it was found in the previous section to predict the liquid temperature more accurately. The liquid temperature distribution along the wall is illustrated in Fig. 12a within this context. It is evident that temperature remains relatively stable and with no substantial change before the final stage of the collapse. At this point, an increase of 25 K is predicted at the time when the shock wave hits the wall. A similar pattern is observed for pressure in Fig. 12b where the wall pressure exceeds 0.4 GPa.

**Fig. 13:**
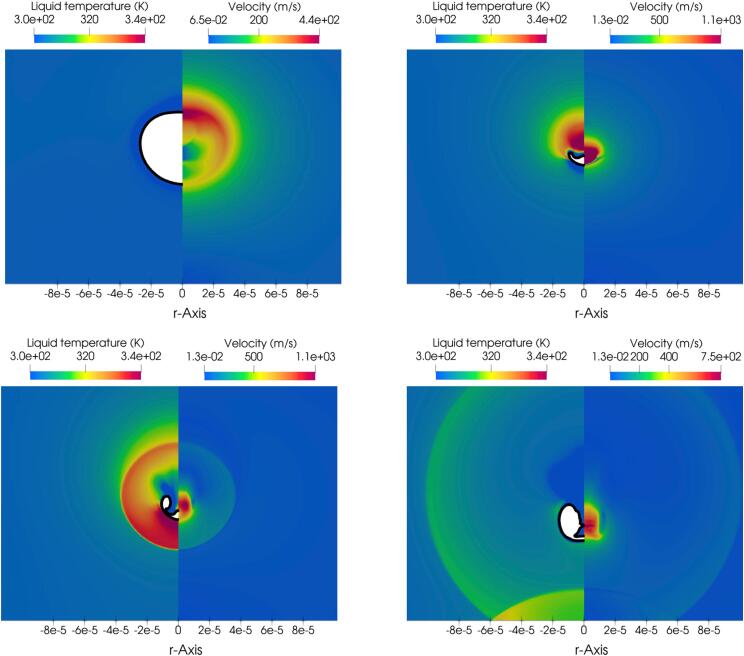
Liquid temperature (left side) and velocity magnitude (right side) at different collapse stages.

In order to gain a deeper insight into the dynamic evolution of liquid temperature, temperature contours are presented in Fig. 13; the plotted temperature values are masked to values up to the maximum value in the liquid phase (thus, the much higher temperatures inside the bubble appear as empty). On the right-hand side of the same plots, the contours depicting the magnitude of the velocity field are plotted.

**Fig. 14:**
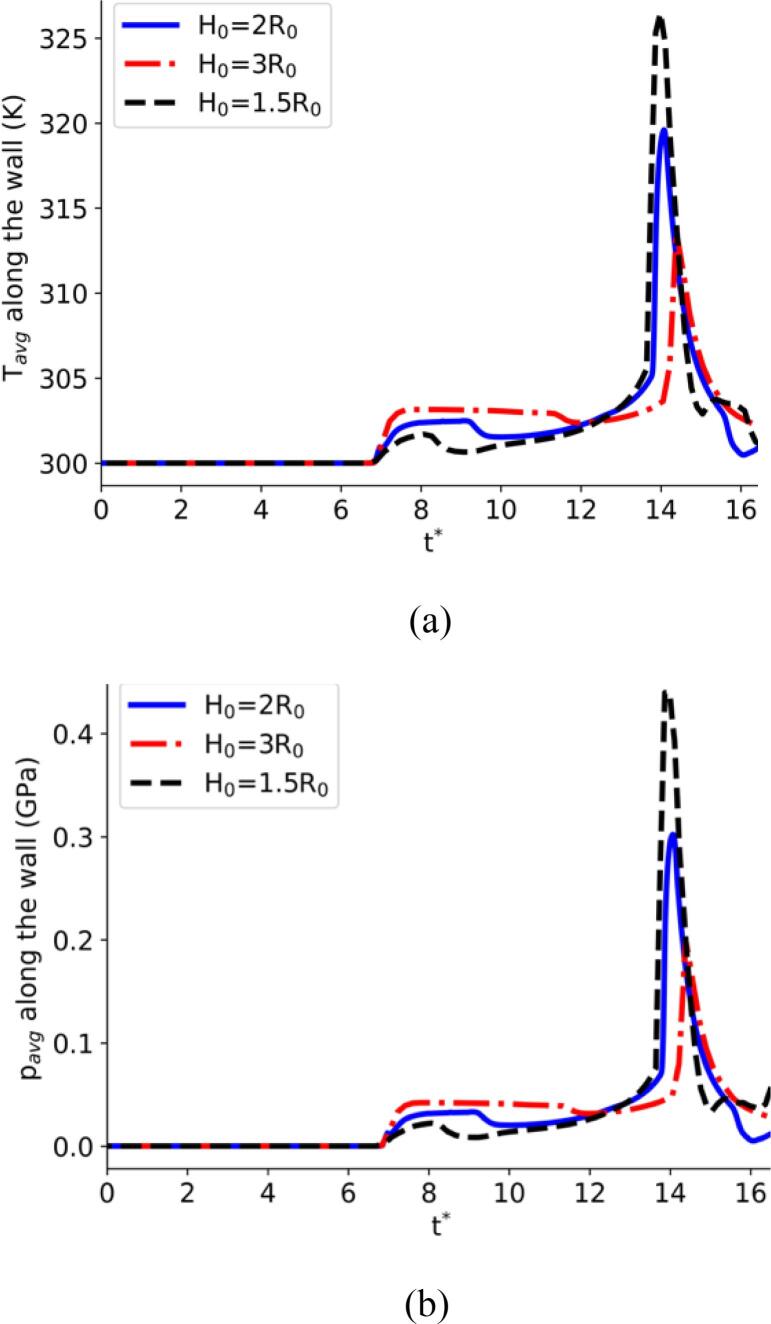
Spaced-averaged (in $r<R_{0}$) liquid temperature (a) and pressure (b) along the wall over time.

According to Fig. 13b it becomes apparent that the liquid temperature can rise significantly to 340 K in the region above the bubble before the collapse. During the rebound, this region cools down while the liquid temperature in the proximity of the bubble is still high, as shown in Fig. 13c. Subsequently, the shock propagates towards the wall where the liquid temperature along the wall increases to 340 K, as depicted in Fig. 13d. Evidently, the shock wave causes this heating rather than the propagation of the pressure pulse of the lithotripter. To further elaborate on this observation, the impact of the initial bubble stand-off distance from the wall on the subsequent rise of liquid temperature have been studied, using various initial stand-off distances. Fig. 14 highlights the temporal evolution of the space-averaged liquid temperature and pressure within the region $r<R_{0}$. As depicted in this figure, a decrease in the initial stand-off distance results in an increase in both the liquid temperature along the wall and the pressure, consistent with the observations reported in [34].

**Fig. 15:**
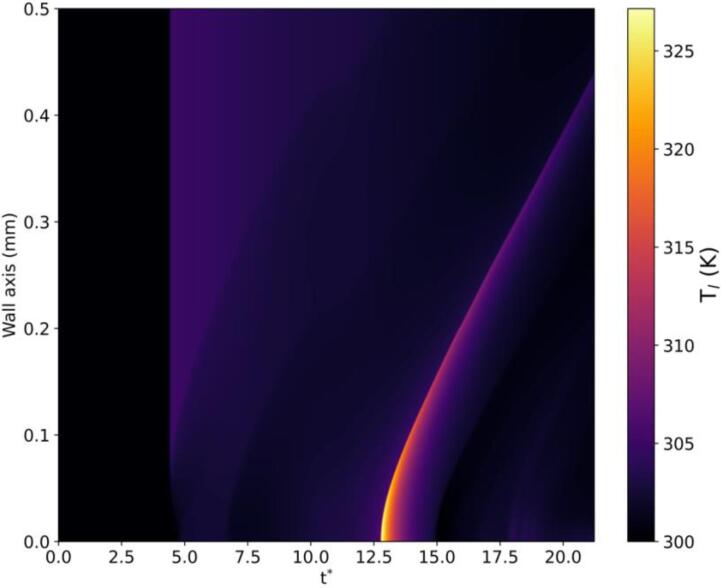
Spatio-temporal change of the liquid temperature along the wall axis.

Finally, the heatmap of the temporal change of the liquid temperature along the rigid wall is depicted in Fig. 15 where the horizontal axis shows the time while the vertical axis represents the rigid wall axis. Accordingly, it is observed that the temperature increase due to the pressure pulse is not significant. However, as the bubble collapses, the maximum temperature raise to T=325 K below the bubble in r≈(0,0.02) mm while tissue damage occurs usually above 325 K [121]. As time progresses, the region exhibiting the highest temperature gradually migrates towards the right boundary, with a reduction in its magnitude.

## 5 Conclusion

In the current study, we expand the Kapila model to incorporate complex EoSs for both the liquid and gas phases. Since real gas effects during bubble collapses were extensively discussed in our former work, the present work has focused on the thermal effects induced in the liquid phase. Particularly, the deficiencies of EoS for the liquid state ought to both the unphysical specific heat ratio and the absence of terms considering repulsive molecular effects was showcased. It was observed that the SG EoS leads to above 800% error in temperature rise compared to the prediction obtained with the IAPWS one, at the highest compression of 10 GPa investigated. Moreover, the more complex and accurate modified Tait and IAPWS EoS were able to resolve and eliminate the spurious liquid temperature front that is predicted when the SG EoS was used, and which can be as high as 400 K. Having demonstrated the ability of the proposed liquid EoS to predict the temperature variation for the benchmark bubble collapse cases, the liquid temperature developing along a solid wall exposed to the violent bubble collapse induced by an ultrasonic pressure pulse simulating that of a commercial lithotripter was studied. Model predictions indicated that the temperature increase during a single collapse event is a function of the bubble initial stand-off distance; shorter initial stand-off distance lead to higher liquid temperatures along the wall.

## Declaration of Competing Interest

The authors declare that they have no known competing financial interests or personal relationships that could have appeared to influence the work reported in this paper.

## Data Availability

The Supporting Information is available free of charge on the ACS Publications website at DOI: 10.25383/city.21262635.
